# Toward Stability Enhancement of NTS_1_R-Targeted Radioligands: Structural Interventions on [^99m^Tc]Tc-DT1

**DOI:** 10.3390/pharmaceutics15082092

**Published:** 2023-08-07

**Authors:** Panagiotis Kanellopoulos, Berthold A. Nock, Eric P. Krenning, Theodosia Maina

**Affiliations:** 1 Molecular Radiopharmacy, INRaSTES, NCSR “Demokritos”, 15341 Athens, Greece; kanelospan@gmail.com (P.K.); nock_berthold.a@hotmail.com (B.A.N.); 2 Cyclotron Rotterdam BV, Erasmus MC, 3015 CE Rotterdam, The Netherlands; erickrenning@gmail.com

**Keywords:** neurotensin subtype 1 receptor, radiolabeled neurotensin, targeted tumor imaging, Tc-99m, metabolic stability, neprilysin, angiotensin-converting enzyme, peptidase-inhibition

## Abstract

The neurotensin subtype 1 receptor (NTS_1_R) is overexpressed in a number of human tumors, thereby representing a valid target for cancer theranostics with radiolabeled neurotensin (NT) analogs like [^99m^Tc]Tc-DT1 (DT1, N_4_-Gly^7^-NT(8-13)). Thus far, the fast degradation of intravenously injected NT–radioligands by neprilysin (NEP) and angiotensin-converting enzyme (ACE) has compromised their clinical applicability. Aiming at metabolic stability enhancements, we herein introduce (i) DT7 ([DAsn^14^]DT1) and (ii) DT8 ([β-Homoleucine^13^]DT1), modified at the C-terminus, along with (iii) DT9 ([(palmitoyl)Lys^7^]DT1), carrying an albumin-binding domain (ABD) at Lys^7^. The biological profiles of the new [^99m^Tc]Tc–radioligands were compared with [^99m^Tc]Tc-DT1, using NTS_1_R-expressing AsPC-1 cells and mice models without or during NEP/ACE inhibition. The radioligands showed enhanced in vivo stability vs. [^99m^Tc]Tc-DT1, with [^99m^Tc]Tc-DT9 displaying full resistance to both peptidases. Furthermore, [^99m^Tc]Tc-DT9 achieved the highest cell internalization and tumor uptake even without NEP/ACE-inhibition but with unfavorably high background radioactivity levels. Hence, unlike C-terminal modification, the introduction of a pendant ABD group in the linker turned out to be the most promising strategy toward metabolic stability, cell uptake, and tumor accumulation of [^99m^Tc]Tc-DT1 mimics. To improve the observed suboptimal pharmacokinetics of [^99m^Tc]Tc-DT9, the replacement of palmitoyl on Lys^7^ by other ABD groups is currently being pursued.

## 1. Introduction

The overexpression of neurotensin subtype 1 receptor (NTS_1_R) has been documented in a number of human cancers, mainly in exocrine pancreatic ductal carcinoma (PDAC) [1,2,3,4], Ewing’s sarcoma [5], and colon [6], prostate [7], and breast cancer [8], and is therefore regarded as a valid biomolecular target for cancer theranostics [9]. Several analogs of the C-terminal hexapeptide fragment of native neurotensin (NT, pyroGlu-Leu-Tyr-Glu-Asn-Lys-Pro-Arg-Arg-Pro-Tyr-Ile-Leu-OH) have been coupled to suitable chelators for stable binding of diagnostic (e.g., Tc-99m, In-111: SPECT imaging; Ga-68: PET imaging) or particle emitters (e.g., Lu-177) for use in the management of NTS_1_R-expressing tumors [10,11,12]. However, results in patients have been suboptimal thus far, a fact attributed to the fast degradation of NT-derived radioligands following their entry in the circulation. Hence, supply to tumor sites and, consequently, tumor-targeting efficacy is compromised [13,14,15,16,17]. Previous studies have revealed the predominant role of two peptidases in the catabolism of NT and its analogs, neprilysin (NEP) and angiotensin-converting enzyme (ACE) [18,19,20,21]. Based on these findings, we recently achieved in situ stabilization of fast biodegradable [^99m^Tc]Tc-DT1 (DT1, N_4_-Gly^7^-NT(8-13); N_4_, 6-(carboxy)-1,4,8,11-tetraazaundecane) and its analogs [22,23] in peripheral mice blood by means of NEP/ACE inhibitors. As expected, tumor uptake in mice was markedly improved, highlighting NEP/ACE-resistance as a crucial feature in the performance of NT–radioligands [24,25].

Translation of the NEP/ACE-inhibition approach in the clinic has to circumvent a number of regulatory challenges related to the administration of two distinct peptidase inhibitors to patients, even though registered drugs for ACE or NEP inhibition are currently available. Thus, lisinopril is a widely used antihypertensive drug with high ACE-inhibition potency [26]. On the other hand, the potent and selective NEP inhibitors thiorphan (released from racecadotril in the anti-diarrhea drug hidrasec [27,28]) or sacubitrilat (released from sacubitril in the anti-hypertensive drug Entresto^®^ [29,30,31]) can be applied following oral administration of hidrasec or Entresto^®^ pills, respectively. It should be noted that hidrasec has been safely and successfully applied in medullary thyroid cancer patients to in situ stabilize a biodegradable gastrin radioligand, thereby indeed improving diagnostic accuracy [32]. Yet, the implementation of double NEP and ACE inhibition in patients, required in the case of NT analogs, undeniably remains a challenging goal. Therefore, we have next directed our efforts in search of [^99m^Tc]Tc-DT1 mimics, which are resistant to at least one of the two peptidases, namely ACE. 

**Figure 1:**
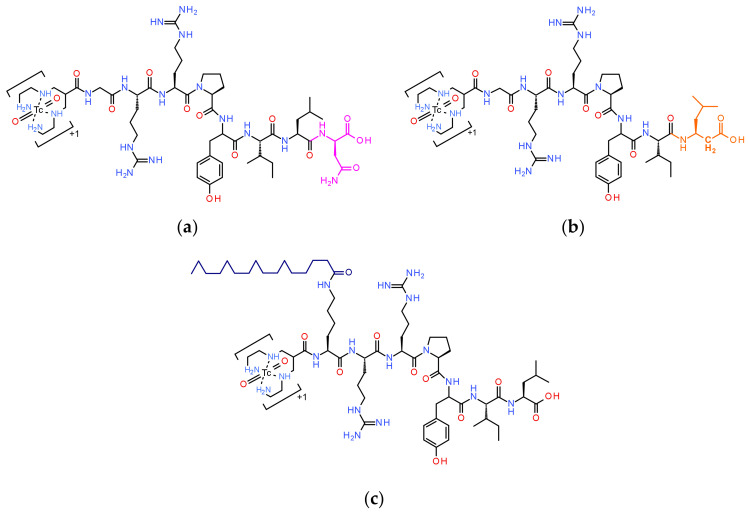
Chemical structures of C-terminal-modified [^99m^Tc]Tc-DT1 mimics (DT1, N_4_-Gly^7^-Arg-Arg-Pro-Tyr-Ile-Leu-OH; N_4_, 6-(carboxy)-1,4,8,11-tetraazaundecane): (**a**) [^99m^Tc]Tc-DT7 (DT7, [DAsn^14^]DT1); (**b**) [^99m^Tc]Tc-DT8 (DT8, ([β-Homoleucine^13^]DT1); and (**c**) [^99m^Tc]Tc-DT9 (DT9, [(palmitoyl)Lys^7^]DT1), carrying a pendant ABD palmitoyl-group at the Lys^7^-linker.

ACE, also known as peptidyl dipeptidase A, primarily cleaves a C-terminal dipeptide from substrates including NT and its analogs [20,21]. For obtaining ACE-resistant [^99m^Tc]Tc-DT1 mimics, we first pursued the Ile^12^ to Tle^12^-replacement route. However, Tle^12^-modified [^99m^Tc]Tc-DT6 (DT6, [N_4_-*β*Ala^7^,Dab^9^,Tle^12^]]NT(8-13)) displayed low internalization efficacy and only slightly improved tumor uptake compared to both [^99m^Tc]Tc-DT5 (DT5, [N_4_-*β*Ala^7^,Dab^9^]]NT(8-13)) and the [^99m^Tc]Tc-DT1 reference [24]. Most importantly, during ACE/NEP inhibition in mice models, tumor uptake of these analogs remained significantly inferior vs. [^99m^Tc]Tc-DT1 [22,23,24,25]. Our next step toward ACE-robust [^99m^Tc]Tc-DT1 mimics was prompted by reports on the resistance of peptide analogs containing D-amino acids at the C-terminus to the hydrolytic action of ACE [33]. Luckily, high-affinity binding of NT(8-13) analogs elongated by a D-amino acid residue at the C-terminus, such as NT(8-13)-DAsn (Ki = 2.2 ± 0.71 nM), was reported [34]. Thus, [^99m^Tc]Tc-DT7 ([DAsn^14^]DT1; Figure 1a) was considered as the first new analog in our study. Another route proposed to stabilize NT–radioligands in human plasma/serum is “homologation” [35,36,37]. Interestingly, replacement of C-terminal Leu^13^ by β-Homoleucine did not impair the binding affinity of [β-Homoleucine^13^]NT(8-13) for the human NTS_1_R (Ki = 3.2 ± 0.75 nM) [37]. Therefore, [^99m^Tc]Tc-DT8 ([β-Homoleucine^13^]DT8; Figure 1b) was selected as the second analog in our study.

As a third analog, we chose [^99m^Tc]Tc-DT9 ([(palmitoyl)Lys^7^]DT1; Figure 1c) based on a different stabilization strategy, namely the introduction of a lipid acid albumin-binding domain (ABD) on the linker. Lipidation of peptides has been often proposed as a useful means to increase metabolic stability and bioavailability [38,39,40,41]. The position of introducing the lipid acid should be carefully selected to avoid interference with receptor binding. In this context, we were intrigued by contulakin-G, a 16-amino-acid peptide (pGlu-Ser-Glu-Glu-Gly-Gly-Ser-Asn-Ala-Thr(R)-Lys-Lys-Pro-Tyr-Ile-Leu-OH; R, the disaccharide beta-D-Galp-(1-->3)-alpha-D-GalpNAc-(1-->); Galp, Galactopyranosyl and GalpNAc, N-acetylgalactosaminepyranosyl) isolated from the venom of the predatory sea snail *Conus geographus*, which displayed binding affinity to the human NTS_1_R [42]. Replacement of the Thr^10^-attached disaccharide by a variety of functional groups, including palmitoyl, led to analogs of improved bioavailability and high resistance to enzymatic degradation [43,44]. Notably, similarly modified NT analogs with improved characteristics were soon developed with pendant groups introduced at the corresponding position 7 in NT [43,44]. These reports provided the rationale for designing [^99m^Tc]Tc-DT9.

In the present work, we compared the biological performance of the newly introduced peptide analogs DT7/8/9 and their [^99m^Tc]Tc–radioligands in NTS_1_R-positive cells and mice models vs. the [^99m^Tc]Tc-DT1 reference, focusing on particular features like metabolic stability, receptor affinity, and internalization capacity. The effects of ACE/NEP inhibition were investigated as well to reveal the best candidates for subsequent evaluation in tumor-bearing mice. Conclusions on the suitability of the two structural intervention approaches on [^99m^Tc]Tc-DT1 were drawn, and further steps to follow are herein discussed. 

## 2. Materials and Methods

### 2.1. Chemicals and Radioligands

#### 2.1.1. Peptides and Protease Inhibitors 

Except for the solvents used in high-performance liquid chromatography (HPLC), which were HPLC grade, all other chemicals used were reagent grade. Neurotensin (NT) was obtained from Bachem (Bubendorf, Switzerland). Entresto^®^ pills (200 mg corresponding to 24 mg/26 mg sacubitril/valsartan per pill; Novartis AG, Basel, Switzerland) were obtained from a local pharmacy. Individual doses were prepared by grinding pills in a mortar to a fine powder, dividing, and suspending in tap water forming a slurry for oral gavage to mice (12 mg/200 μL per animal) [24,25]. The ACE inhibitor lisinopril (Lis, lisinopril dehydrate, ((S)1–1-[N2-(1-carboxy-3-phenylpropyl)-lysyl-proline dehydrate, MK 521) was provided by Sigma-Aldrich (St. Louis, MO, USA).

The peptide conjugates DT1 (N_4_-Gly-Arg-Arg-Pro-Tyr-Ile-Leu-OH, N_4_ = 6-(carboxy)-1,4,8,11-tetraazaundecane; reference), DT7 (N_4_-Gly-Arg-Arg-Pro-Tyr-Ile-Leu-DAsn-OH), DT8 (N_4_-Gly-Arg-Arg-Pro-Tyr-Ile-β-Homoleucine-OH; β-Homoleucine = 3-amino-5-methylhexanoic acid), and DT9 (N_4_-(palmitoyl)Lys-Arg-Arg-Pro-Tyr-Ile-Leu-OH; palmitoyl = hexadecanoyl) were purchased from PiChem Forschungs- und Entwicklungs GmbH (Raaba-Grambach, Austria); chemical structures are presented in Figure 1. Analytical data, including purity determined by HPLC analysis and matrix-assisted laser desorption/ionization–time of flight (MALDI-TOF) mass spectrometry (MS) findings, is summarized in Table S1.

Technetium-99m, used for labeling, was eluted as [^99m^Tc]NaTcO_4_ in normal saline from a [^99^Mo]Mo/[^99m^Tc]Tc generator (Ultra-Technekow V4 Generator, Curium Pharma, Petten, The Netherlands). For preparation of [^125^I]I-Tyr^3^-NT, [^125^I]NaI in dilute sodium hydroxide solution (pH 8–11) was obtained from Perkin Elmer (Waltham, MA, USA).

#### 2.1.2. Radiolabeling

The peptide conjugates were dissolved at 2 mg/mL in doubly distilled H_2_O and were stored in 50 μL aliquots in Eppendorf Protein LoBind tubes at −20 °C. Labelling was performed in a LoBind Eppendorf tube containing phosphate buffer (0.5 M, pH 11.5, 50 μL) to which [^99m^Tc]NaTcO_4_ (420 μL generator eluate) was added, followed by sodium citrate (0.1 M, 5 μL), the peptide stock solution (15 μL, 15 nmol), and SnCl_2_ freshly dissolved in EtOH (10 μL, 10 μg). The mixture was incubated for 30 min at room temperature (RT), and the pH was adjusted to 7.4 using 0.1 M HCl.

For the quality control, HPLC and instant thin-layer chromatography (iTLC) were applied. HPLC analyses were conducted on a Waters Chromatograph coupled to a 2998-photodiode array UV detector (Waters, Vienna, Austria) and a Gabi gamma detector (Raytest RSM Analytische Instrumente GmbH, Straubenhardt, Germany). Data acquisition and processing were achieved by the Empower Software 3.0 (Waters, Milford, MA, USA). A Symmetry Shield RP-18 (5 μm, 3.9 mm × 20 mm) cartridge column (Waters, Eschborn, Germany) was eluted with a flow rate of 1 mL/min with a linear gradient (system 1): from 100% A/0% B to 60% A/40% B in 20 min (A: 0.1% aqueous TFA and B: MeCN). For [^99m^Tc]Tc-DT9, analyses were performed on an XTerra RP-8 (5 μm, 3.9 mm × 20 mm) cartridge column (Waters, Vienna, Austria) eluted with a with a flow rate of 1 mL/min linear gradient (system 1b): from 60% A/40% B to 20% A/80% B in 20 min (A: 0.1% aqueous TFA supplemented with 10 mM 1-Heptanesulfonic acid sodium salt; B: MeCN/0.1% TFA 8/2, supplemented with 10 mM 1-Heptanesulfonic acid sodium salt). For iTLC, Whatman 3 mm chromatography paper strips (GE Healthcare, Chicago, IL, USA) were developed up to 10 cm from the point of origin with 5 M NH_4_AcO/MeOH 1:1 (*v*/*v*) as mobile phase for the detection of reduced, hydrolyzed technetium (R_f_ = 0 cm) or acetone for the detection of free unreduced [^99m^Tc]TcO_4_^−^ (R_f_ = 10 cm). Sample radioactivity was measured in a γ-counter (automated multi-sample, well-type instrument with a NaI(Tl) 3″ crystal, Canberra Packard Cobra^TM^ Quantum U5003/1, Auto-Gamma^®^ counting system; Canberra Packard, Ramsey, MN, USA). Radioligand samples used in all biological experiments were prepared in a phosphate-buffered saline (PBS, pH 7.4)/EtOH *v*/*v* 9/1 solution and tested before and after completion of all experiments. In the case of [^99m^Tc]Tc-DT9, this solution additionally contained 0.1% Tween-80 (Sigma-Aldrich Inc., St. Louis, MO, USA) to combat sticking to plastic and glass containers.

For I-125 labeling of NT, the chloramine T method was applied based on a published protocol [45]. Separation of [^125^I]I-Tyr^3^-NT from the reaction mixture was achieved by RP-HPLC on a Symmetry Shield RP-18 (5 μm, 3.9 mm × 150 mm) cartridge column (Waters, Eschborn, Germany) eluted with a flow rate of 1 mL/min with the following gradient: from 100% A/0% B to 80% A/20% B in 5 min and then to 70% A/30% B in 40 min (A: 0.1% TFA/0.05% Et_3_N (pH 2–2.5); B: 0.1% TFA/0.05% Et_3_N in MeCN). Elution time (*t*_R_) [^125^I]I-Tyr^3^-NT: 28 min ([^125^I]I-Tyr^11^-NT: 29 min, NT: 20.5 min). A stock solution of purified [^125^I]I-Tyr^3^-NT in 0.1% BSA-PBS buffer was kept in aliquots at −20 °C for use in competition binding assays (molar activity of 74 GBq/μmol).

All procedures involving radioactive materials were performed by trained and authorized personnel using suitable shielding in licensed laboratories complying with European radiation-safety guidelines and supervised by the Greek Atomic Energy Commission (license # A/435/17092/2019).

### 2.2. Cell Studies

#### 2.2.1. Cell Culture

The two human NTS_1_R-expressing cell lines, the colorectal adenocarcinoma WiDr (LGC Promochem; Teddington, UK) and the pancreatic adenocarcinoma AsPC-1 cell lines (LGC Standards GmbH; Wesel, Germany), were used in this study. The WiDr cells were grown in McCoy’s GLUTAMAX-I medium and the AsPC-1 cells in Roswell Park Memorial Institute-1640 (RPMI), both supplemented with 10% (*v*/*v*) fetal bovine serum (FBS), 100 U/mL penicillin, and 100 µg/mL streptomycin. Cells were cultured in 75 cm^2^ flasks at 37 °C (95% humidity, 5% CO_2_) in a Heal Force SMART CELL HF-90 incubator (Shanghai, China) and split at 80–90% confluency applying a Trypsin/EDTA (0.05%/0.02% *w*/*v*) solution. Culture media were obtained from Gibco BRL, Life Technologies (Grand Island, NY, USA) and supplements as well as the Trypsin/EDTA solution from Biochrom KG Seromed (Berlin, Germany).

#### 2.2.2. Competition Binding Experiments

Cell membrane homogenates from WiDr cells were collected and stored in Tris/EDTA (10 mM Tris, 0.1 mM EDTA, pH 7.4) solution at −80 °C, as previously described. On the day of the experiment, the aliquots thereof were thawed, combined, and diluted in cold binding buffer (BB: 50 mM HEPES, 5.5 mM MgCl_2_, 0.1 mg/mL bacitracin, 1% *w*/*v* BSA, pH 7.4). A dilution series of each test peptide (10^−12^–10^−5^ M) and a fresh solution of [^125^I]I-Tyr^3^-NT (~40,000 cpm/70 μL, 214 pM) were prepared and kept on ice. In each RIA tube per placed on ice (in triplicate for each concentration point), the following ice-cold solutions were added: test peptide solution (30 μL), the [^125^I]I-Tyr^3^-NT radioligand (70 μL), and membrane homogenate (200 μL). Tubes were incubated under constant stirring for 1 h at 22 °C in an Incubator-Orbital Shaker (MPM Instr. Srl; Bernareggio, Italy). The incubation was terminated by placing the tubes on ice and adding ice-cold washing buffer (10 mM HEPES, 150 mM NaCl, pH 7.4). Samples were rapidly passed through Whatman GF/B filters (presoaked for 1 h in BB) on a 48-sample Brandel Cell Harvester (Adi Hassel Ingenieur Büro, Munich, Germany). Separate filters were measured for radioactivity on the γ-counter, and the half maximal inhibition concentration (IC_50_) was calculated applying a non-linear one-site model (GraphPad Prism Software 6.0, San Diego, CA, USA). Results represent average IC_50_ values ± standard deviation (sd), n = 3.

#### 2.2.3. Internalization in AsPC-1 Cells 

The NTS_1_R-specific internalization of [^99m^Tc]Tc-DT1/7/8/9 radioligands was compared in AsPC-1 cells. The cells were seeded in 6-well plates (1 × 10^6^ cells per well) and left to grow overnight. The following day, the medium was aspirated, and the cells were washed twice with internalization medium (2 mL IM: RPMI supplemented with 1% *v*/*v* FBS) on ice. They were placed on the bench, and warm IM (1200 μL 37 °C) was added per well, followed by radioligand solution (150 μL, 250 fmol) and either IM (150 μL total: T; upper wells) or NT (10^−5^ M in IM, non-specific: NS; lower wells). The plates were placed in the Incubator-Orbital Shaker at 37 °C for 1 h, and the incubation was interrupted by placing the plates on ice. The supernatant was collected in RIA tubes, and the cells were washed with 1 mL phosphate-buffered saline (PBS, pH 7.4, 4 °C) containing 0.5% *w*/*v* BSA, and the washing was also collected in the same RIA tube. Cells were treated twice for 5 min with glycine buffer (600 μL, 50 mM glycine, 0.1 M NaCl, pH 2.8), and supernatants were collected (membrane bound fraction, MB). The cells were washed again with 1 mL ice-cold PBS-BSA buffer, which was discarded. Finally, cells were lysed (2 × 600 μL 1 M NaOH), and the lysates were collected (internalized fraction, IF). Sample radioactivity was measured on the γ-counter. Specific values for MB and IF were acquired by subtracting NS values from T ones. Results are expressed as mean percentage of added radioactivity ± sd, n = 3. In addition, time-dependent internalization in AsPC-1 cells was directly compared for [^99m^Tc]Tc-DT1/9 by incubation at 37 °C for 15 min, 30 min, 1 h, and 2 h, following the above-described protocol for each time point. 

### 2.3. Animal Studies

#### 2.3.1. Stability Studies

For the assessment of in vivo stability of [^99m^Tc]Tc-DT7/8/9, healthy Swiss Albino mice (33 animals, >8 weeks of age, body weight: 30 ± 5 g) were obtained from NCSR “Demokritos” Animal House (Athens, Greece). Animals were injected through the tail vein with the radioligand (100 μL, 2 nmol in vehicle: PBS/EtOH 9/1 *v*/*v*, with the addition of 0.1% Tween-80 in the case of [^99m^Tc]Tc-DT9) plus vehicle (100 μL; controls) or plus Lis (100 μg in 100 μL vehicle; Lis). Additional mice groups received per os a suspension of Entresto^®^ (200 μL, 12 mg, 30 min prior to the i.v. injection of the radioligand plus vehicle—Entresto^®^ group; or the i.v. injection of the radioligand plus Lis—Entresto^®^+Lis group). Animals were euthanized 5 min post injection (pi), and blood samples were drawn from the heart via a prechilled insulin syringe and transferred rapidly into pre-cooled 1.5 mL LoBind Eppendorf tubes containing EDTA (20 μL, 0.1 mM EDTA), and the collected radioactivity was measured in a dose calibrator (CURIEMENTOR 4, PTW Freiburg-GmbH; Freiburg, Germany). The tubes were centrifuged for 10 min at 2000× *g* at 4 °C in a Hettich Universal 320 R centrifuge (Tuttlingen, Germany). The plasma was collected, diluted in a 1:1 *v*/*v* ratio with MeCN, and thoroughly mixed; sample tubes were again centrifuged for 10 min at 15000× *g* at 4 °C. The supernatant was collected and the volume reduced to 50–100 μL under a gentle flux of N_2_ and mild heating at 60 °C. Samples were diluted in physiological saline to a final volume of 450–500 μL and filtered through Millex GV filters (0.22 μm, 13 mm diameter, Millipore; Milford, CT, USA). Sample activity was measured in the dose calibrator, and aliquots were analyzed by radio-HPLC on a Symmetry Shield RP18 cartridge column (5 μm, 3.9 mm × 20 mm; Waters, Eschborn, Germany) eluted at a flow rate of 1 mL/min with the linear gradient (system 2): from 100% A/0% B to 60% A/40% B in 40 min (A: 0.1% aqueous TFA; B: MeCN). For [^99m^Tc]Tc-DT9, analyses were performed on an XTerra RP-8 (5 μm, 3.9 mm × 20 mm) cartridge column (Waters, Vienna, Austria) eluted with a with a flow rate of 1 mL/min linear gradient (system 2b): from 90% A/10% B to 0% A/100% B in 45 min (A: 0.1% aqueous TFA supplemented with 10 mM 1-Heptanesulfonic acid sodium salt; B: MeCN/0.1% TFA 8/2, supplemented with 10 mM 1-Heptanesulfonic acid sodium salt). The *t*_R_ of intact [^99m^Tc]Tc-DT7, [^99m^Tc]Tc-DT8, or [^99m^Tc]Tc-DT9 was determined by co-injection of blood samples processed as above with an aliquot of the labeling solution. Results were obtained from three mice per analog per treatment and are presented as average percentage of intact radiopeptide ± sd.

#### 2.3.2. Biodistribution of [^99m^Tc]Tc-DT9 in SCID Mice Bearing AsPC-1 Xenografts

Twenty male severe combined immunodeficiency (SCID) mice (23.1 ± 1.6 g body weight, six weeks of age on arrival day; NCSR “Demokritos” Animal House, Athens, Greece) were used in the biodistribution experiments. Animals were subcutaneously inoculated in their right flanks with a sterile suspension of freshly harvested AsPC-1 cells (150 μL, 5 × 10^6^ cells/animal), and 3–4 weeks later, they developed well-palpable tumors at the implantation sites. During this period, mice were housed in suitable facilities under sterile conditions with 12 h day/night cycles and were provided with sterilized chow food and drinking water ad libitum. At the date of the experiment, animals were randomly divided in groups of four and received through the tail vein a bolus of [^99m^Tc]Tc-DT9 (100 μL, 3 pmol in vehicle: PBS/EtOH 9/1 *v*/*v* with the addition of 0.1% Tween-80) plus vehicle (100 μL; controls at 4 and 24 h pi) or plus Lis (100 μg in 100 μL vehicle); the latter groups had additionally received per os 30 min in advance Entresto^®^ (200 μL, 12 mg; Entresto^®^+Lis groups at 4 and 24 h pi). A further 4 h group of animals was treated with Entresto^®^, and 30 min later, mice were co-injected with excess NT and Lis (100 μg NT and 100 μg Lis in 100 μL vehicle—NTS_1_R block). At the predetermined time intervals, mice were euthanized and weighted, and their blood, organs, and tissue samples of choice as well as the implanted tumors were collected and weighted. Sample radioactivity was measured on the γ-counter together with proper standards of the injected dose. Results were calculated as percentage of injected activity per gram tissue (%IA/g) and provided as mean %IA/g values ± sd. For comparisons, a two-way ANOVA with Tukey’s post hoc analysis was adopted (PRISM^TM^ 6.0 GraphPad Software, San Diego, CA, USA). *p*-values < 0.05 were considered statistically significant.

Mice experiments were conducted in licensed facilities (EL 25 BIO exp021) and complied with European and national regulations. The study protocols were approved by the Department of Agriculture and Veterinary Service of the Prefecture of Athens (#1609, 24-04-2019 for the stability studies and #1610, 24-04-2019 for the biodistribution studies)

## 3. Results

### 3.1. Ligands and Radioligands

Analytical data for the new DT7, DT8, and DT9 bioconjugates are presented in Table S1. HPLC analytical data in two separate systems and the MALDI-TOF MS results are consistent with the formation of the desired peptide conjugates in high purity (≥99%). 

Labeling of DT1/7/8/9 with Tc-99m was easily concluded at ambient temperature using SnCl_2_ as reductant and sodium citrate as transfer ligand in alkaline aqueous medium, as previously reported [24,25]. Molar activities of 20–40 MBq/nmol peptide typically led to >98% radiochemical purities, according to radioanalytical HPLC (single radiopeptide species) and iTLC findings (sum of radiochemical impurities [^99m^Tc]TcO_4_^−^, [^99m^Tc]citrate, and [^99m^Tc]TcO_2_ × nH_2_O <2%). Therefore, radiolabeled analogs were used as such in all further experiments. 

### 3.2. In Vitro Evaluation

#### 3.2.1. Binding Affinity for the Human NTS_1_R

**Figure 2:**
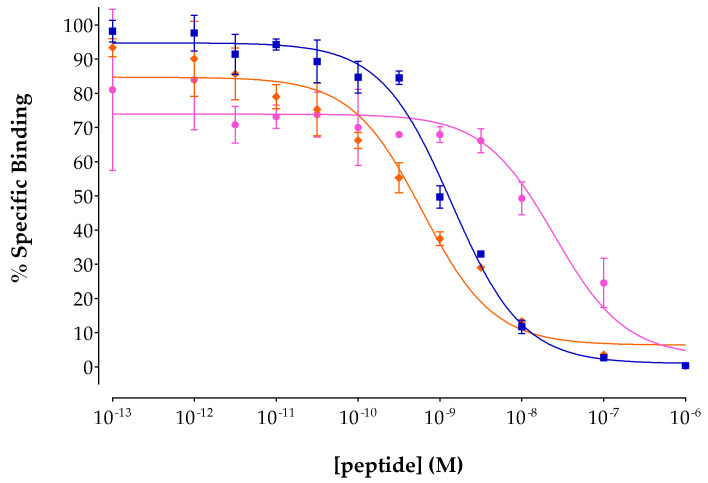
Displacement of [^125^I]I-Tyr^3^-NT from NTS_1_R binding sites in WiDr cell membranes by increasing concentrations of: DT7, pink line (IC_50_ = 29.05 ± 4.11 nM, n = 4); DT8, orange line (IC_50_ = 0.63 ± 0.05 nM, n = 3); DT9, blue line (IC_50_ = 0.89 ± 0.70 nM, n = 4). results represent mean IC_50_ values ± sd; n, number of separate experiments in triplicate.

The affinities of the new peptide conjugates DT7/8/9 for the human NTS_1_R were determined during competition binding assays in freshly harvested WiDr cell membranes, using [^125^I]I-Tyr^3^-NT as the radioligand. As shown in Figure 2, the analogs displaced [^125^I]I-Tyr^3^-NT from the NTS_1_R-sites on the membranes in a monophasic and concentration-dependent manner and can be ordered in the following rank of declining affinity: DT8 (IC_50_ = 0.63 ± 0.05 nM, n = 3) ≥ DT9 (IC_50_ = 0.89 ± 0.70 nM, n = 4; *p* > 0.05) >> DT7 (IC_50_ = 29.05 ± 4.11 nM, n = 4; *p* > 0.0001). The sub-nanomolar values determined for DT8 and DT9 demonstrate that neither the C-terminal Leu^13^/β-Homoleucine^13^-replacement nor the (palmitoyl)Lys^7^-modification of the DT1 motif markedly affected the affinity for the NTS_1_R compared to unmodified DT1 (IC_50_ = 0.14 ± 0.01 nM, n = 4; *p* > 0.05) [22]. In contrast, the C-terminal elongation of DT1 by DAsn^14^ in the case of DT7 provoked a drop of binding affinity to the NTS_1_R by a power of 100 (*p* > 0.0001).

#### 3.2.2. Radioligand Internalization in AsPC-1 Cells

**Figure 3:**
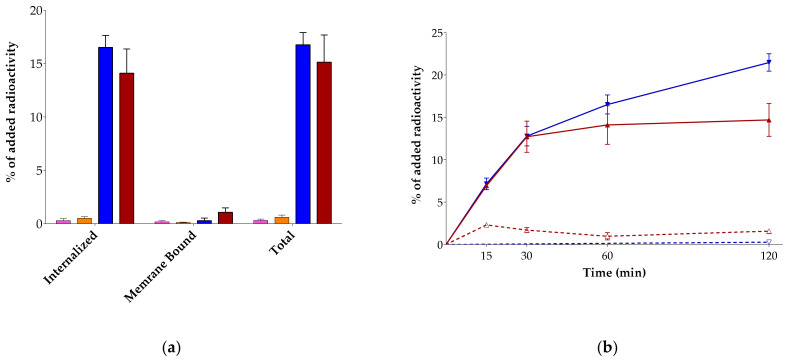
(**a**) NTS_1_R-specific uptake (internalized + membrane bound = total) of [^99m^Tc]Tc-DT7 (pink bars), [^99m^Tc]Tc-DT8 (orange bars), [^99m^Tc]Tc-DT9 (blue bars), and [^99m^Tc]Tc-DT1 (reference; red bars) in AsPC-1 cells as confluent monolayers during 1 h incubation at 37 °C. (**b**) Time-dependent internalization of [^99m^Tc]Tc-DT9 (blue lines) and [^99m^Tc]Tc-DT1 (reference; red lines) in AsPC-1 cells; solid lines correspond to internalized and dotted lines to membrane bound fractions. Results represent average values ± sd (n = 3, in triplicate).

The uptake and internalization of [^99m^Tc]Tc-DT7/8/9 was directly compared with the [^99m^Tc]Tc-DT1 reference during 1 h incubation at 37 °C in AsPC-1 cells [25]. Results of NTS_1_R-specific uptake and internalization are summarized in Figure 3a and reveal that [^99m^Tc]Tc-DT7/8 failed to internalize and accumulate in the cells compared with the [^99m^Tc]Tc-DT1 reference ([^99m^Tc]Tc-DT7: 0.30 ± 0.13%, [^99m^Tc]Tc-DT8: 0.6 ± 0.2%-specific cell uptake of added radioactivity vs. [^99m^Tc]Tc-DT1: 15.15 ± 2.54%-specific cell uptake of added radioactivity at 1 h; *p* < 0.0001). On the other hand, [^99m^Tc]Tc-DT9 displayed a trend for higher internalization (16.53 ± 1.12% specific of added radioactivity) and cell uptake (16.76 ± 1.16% specific of added radioactivity) compared with [^99m^Tc]Tc-DT1 (14.11 ± 2.28% *p* > 0.05 and 15.15 ± 2.54% *p* > 0.05, respectively). The internalization rates of [^99m^Tc]Tc-DT9 and [^99m^Tc]Tc-DT1 were compared at the 15, 30, 60, and 120 min time points, and the results are shown in Figure 3b. According to these findings, [^99m^Tc]Tc-DT9 and [^99m^Tc]Tc-DT1 displayed comparable internalization rates between 15 min and 30 min incubation at 37 °C in AsPC-1 cells, but [^99m^Tc]Tc-DT9 started displaying superior internalization at 1 h (see above; *p* > 0.05), which became significantly higher at 2 h ([^99m^Tc]Tc-DT9: 21.5 ± 1.0% vs. [^99m^Tc]Tc-DT1: 14.7 ± 1.9%-specific uptake of added radioactivity; *p* < 0.001). In all cases, radioligands were taken up by AsPC-1 cells via an NTS_1_R-mediated process, as indicated by the lack of uptake observed in the presence of excess NT (results not included here). Furthermore, the bulk of cell-bound radioactivity for all radioligands was found in the internalized fraction, as is consistent with a radio-agonist profile.

### 3.3. Animal Studies

#### 3.3.1. Radioligand Metabolic Stability in Mice

**Figure 4:**
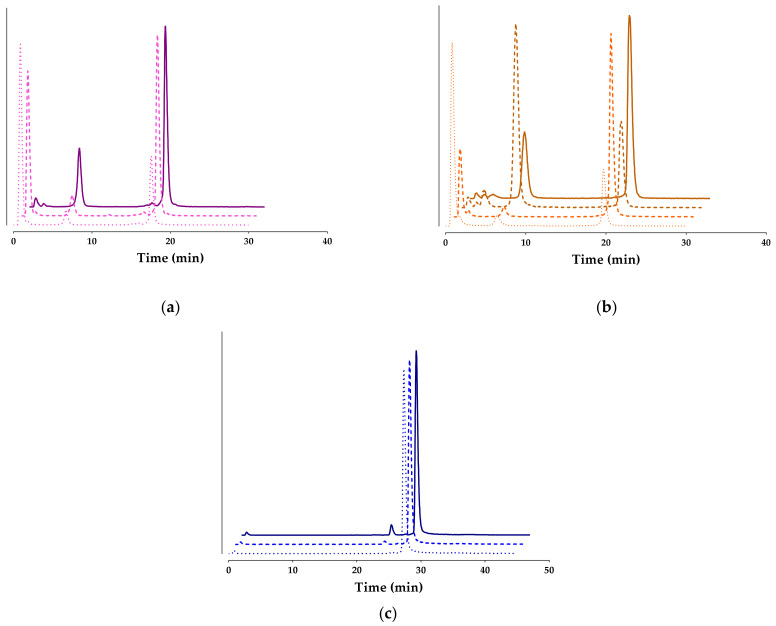
Representative radiochromatograms of HPLC analysis of mouse blood samples collected 5 min pi of (**a**) [^99m^Tc]Tc-DT7 (pink lines), (**b**) [^99m^Tc]Tc-DT8 (orange lines), or (**c**) [^99m^Tc]Tc-DT9 (blue lines) administered without treatment (controls **…….**) or treated with Entresto^®^ (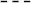) or with Lis (darker 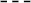) or with the Entresto^®^+Lis combination (darker solid lines 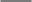; HPLC system 2); percentages of intact radioligand are summarized in Table 1.

The radioligand metabolic stability was compared in peripheral mice blood at 5 min pi in groups of animals without any treatment (controls) or mice treated with Entresto^®^, with in vivo releasing of the NEP inhibitor or the ACE inhibitor Lis, or their combination (Entresto^®^+Lis), as described in the experimental section and applying radio-HPLC. Representative radiochromatograms are shown in Figure 4 for [^99m^Tc]Tc-DT7, [^99m^Tc]Tc-DT8, and [^99m^Tc]Tc-DT9, while cumulative results are presented in Table 1, including findings previously reported for [^99m^Tc]Tc-DT1 for easy comparison purposes [24,25]. 

It is interesting to observe that the structural interventions on the [^99m^Tc]Tc-DT1 motif significantly increased the metabolic stability of all new analogs albeit to a different extent. Thus, the C-terminal modifications in [^99m^Tc]Tc-DT7 and [^99m^Tc]Tc-DT8 increased the stability by more than 200-fold, but only [^99m^Tc]Tc-DT9 achieved clear resistance to both NEP and ACE. As a result, treatment of mice with Entresto^®^ or Entresto^®^+Lis had no effect on the stability of [^99m^Tc]Tc-DT9 in peripheral mice blood at 5 min pi (*p* > 0.05 amongst these animal groups). In the case of [^99m^Tc]Tc-DT7 and [^99m^Tc]Tc-DT8 though, treatment of mice with Entresto^®^ alone resulted in pronounced increases in metabolic stability (56.61 ± 7.92 vs. 26.91 ± 1.91 in controls, *p* < 0.0001; 60.72 ± 8.35 vs. 20.68 ± 3.10 in controls, *p* < 0.0001, respectively). Interestingly, treatment of mice with the Entresto^®^+Lis combination further increased these values but not significantly (*p* > 0.05), a finding consistent with a predominant role of NEP in the catabolism of these particular analogs. In contrast, [^99m^Tc]Tc-DT1 was exposed to the combined action of NEP and ACE (1.81 ± 0.77 in controls vs. 5.46 ± 3.86 in the Entresto^®^ group, *p* < 0.0001; vs. 18.77 ± 2.54 in the Lis group, *p* < 0.0001; vs. 63.80 ± 7.51 in the Entresto^®^+Lis combination group; *p* < 0.0001).

#### 3.3.2. Biodistribution of [^99m^Tc]Tc-DT9 in Mice Bearing AsPC-1 Xenografts 

**Figure 5:**
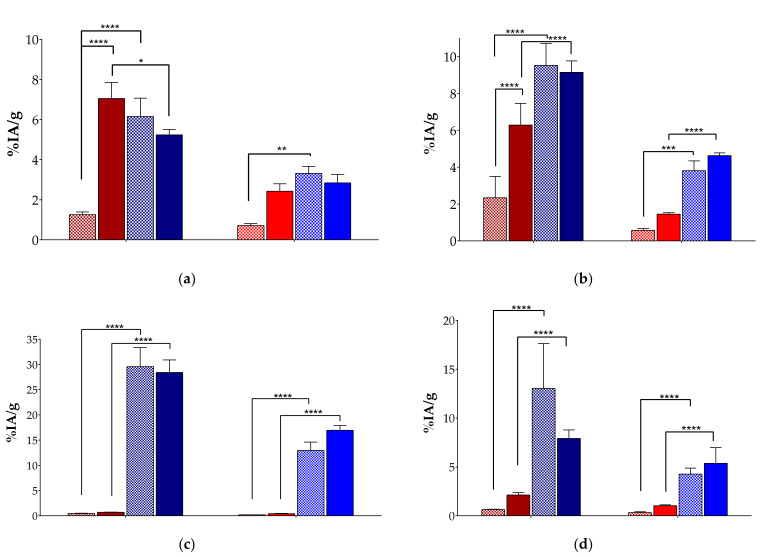
Comparative biodistribution data of [^99m^Tc]Tc-DT9 (blue bars) and [^99m^Tc]Tc-DT1 (reference; red bars) in SCID mice bearing AsPC-1 xenografts at 4 h (controls—dark checkered bars; Entresto^®^+Lis treated—dark solid bars) and 24 h pi (controls—light checkered bars; Entresto^®^+Lis treated—light solid bars) for (**a**) AsPC-1 tumors and (**b**) kidneys, (**c**) liver, and (**d**) intestines; data are expressed as %IA/g and represent average values ± sd, n = 4; statistically significant differences between treatments and radioligands at 4 and 24 h pi: ****: *p* < 0.0001, ***: *p* < 0.001, **: *p* < 0.01, and *: *p* < 0.05.

The biodistribution results of [^99m^Tc]Tc-DT9 in SCID mice bearing subcutaneous AsPC-1 tumors are summarized for 4 and 24 h pi in Table 2. Animals include subgroups without or after treatment with the Entresto^®^+Lis combination for both time points; for the 4 h pi interval, a further group additionally received excess i.v. NT for in vivo NTS_1_R blockade. Results are expressed as mean %IA/g ± sd (n = 4). Selected biodistribution data for AsPC-1 tumors, kidneys, liver, and intestines, including both untreated and Entresto^®^+Lis treated subgroups, of [^99m^Tc]Tc-DT9 are compared vs. [^99m^Tc]Tc-DT1 at 4 and 24 h pi and their statistically significant differences indicated in Figure 5. 

It is interesting to note that the uptake of [^99m^Tc]Tc-DT9 in the AsPC-1 tumors in controls is significantly higher than that of [^99m^Tc]Tc-DT1 (6.15 ± 0.92%IA/g vs. 1.25 ± 0.14%IA/g; *p* < 0.0001) at 4 h pi, most probably a result of its higher in vivo stability. This hypothesis is further supported by the lack of significant change in the tumor uptake of [^99m^Tc]Tc-DT9 between controls and the Entresto^®^+Lis group of animals (6.15 ± 0.92%IA/g vs. 5.24 ± 0.27%IA/g; *p* > 0.05) at 4 h pi. Co-injection of excess NT to in vivo block the NTS_1_R sites on the tumor caused a drop of uptake (6.15 ± 0.92%IA/g vs. 3.68 ± 0.92%IA/g; *p* < 0.01), which is in line with a receptor-mediated process. However, the observed reduction was far below the 90% previously reported for other NT-based radioligands [24,25], a finding assigned to the exceptionally high radioactivity levels found in the blood of all animal groups at 4 h pi (>4%IA/g vs. 0.07 ± 0.01%IA/g of [^99m^Tc]Tc-DT1 in controls; *p* < 0.0001 and 0.08 ± 0.02%IA/g in the Entresto^®^+Lis group; *p* < 0.0001). Notably, the tumor uptake of [^99m^Tc]Tc-DT9 at 24 h pi remained significantly higher than [^99m^Tc]Tc-DT1 in controls (3.32 ± 0.35%IA/g vs. 0.71 ± 0.10%IA/g, respectively; *p* < 0.01), emphasizing the impact of metabolic stability at early time points on tumor uptake at much later time intervals. Interestingly, a statistically significant difference was not observed in the treated groups of animals at 24 h pi (*p* > 0.05).

High uptake and retention was observed for [^99m^Tc]Tc-DT9 in most tissues of the body and especially in the kidneys, the liver, and intestines, which surpassed by far that of [^99m^Tc]Tc-DT1 (Figure 5). For example, the uptake of [^99m^Tc]Tc-DT9 at 4 h pi was markedly increased compared with the unmodified reference in the liver (29.56 ± 3.77%IA/g vs. 0.44 ± 0.05%IA/g in controls; *p* < 0.0001) and intestines (13.02 ± 4.60%IA/g vs. 0.65 ± 0.04%IA/g in controls; *p* < 0.0001). This pronounced and persisting uptake could be partially attributed to the high blood levels of the ABD-modified radioligand combined with the high lipophilicity of the pendant palmitoyl group, resulting in an overall unfavorable in vivo profile for further clinical translation.

## 4. Discussion

The performance of radioligands based on the NT(8-13) motif has been largely compromised by suboptimal metabolic stability following their entry into circulation. In this respect, they are exposed to the rapid hydrolytic action of two major peptidases, ACE and NEP, with which they come into contact on their way to the NTS_1_R target, while evading degradation by peptidases compartmentalized within cells, such as EC 3.4.24.15 (TOP, thimet-oligopeptidase, cleaving the Arg^8^–Arg^9^ bond) or EC 3.4.24.16 (neurolysin, hydrolyzing the Pro^10^–Tyr^11^ bond) [18,20,21,24,46,47,48]. The peptidyl dipeptidase ACE hydrolyzes NT and its analogs at the Tyr^11^–Ile^12^ bond, whereas the endopeptidase NEP cleaves both the Pro^10^–Tyr^11^ and the Tyr^11^–Ile^12^ bonds [18,19,20,21]. In the field of nuclear medicine, most metabolic stability assessments are typically performed by analysis of radioligand incubates in plasma or serum. While ACE is present in considerable levels in these biological fluids [17,33,49], NEP is scarcely present, and thus, its degrading role was inadvertently overlooked till recently [24,25,28,50]. Thus, efforts were initially directed at the stabilization of the ACE-hydrolyzed Tyr^11^–Ile^12^ bond, predominantly via Ile^12^ substitution by Tle^12^ [17,23]. 

Following this route, we have indeed observed metabolic stability enhancement in mice plasma incubates and NTS_1_R-mediated tumor uptake in mice in the case of [^99m^Tc]Tc-DT6, a Tle^12^ [^99m^Tc]Tc-DT1 mimic, compared with [^99m^Tc]Tc-DT5 [23]. On these grounds, [^99m^Tc]Tc-DT6 was selected for diagnostic imaging of NTS_1_R-expressing tumors in human but proved unsuccessful in this respect [14]. We were able to recently demonstrate that [^99m^Tc]Tc-DT6, albeit less degraded in peripheral mice blood than [^99m^Tc]Tc-DT1 (55.1 ± 3.9% vs. 1.8 ± 0.8% intact at 5 min pi; *p* < 0.0001), reached full metabolic stability only during in situ inhibition of NEP (to 89.3 ± 6.7% intact; *p* < 0.0001), revealing NEP as the major catabolizing peptidase [24]. Furthermore, the Tle^12^ substitution impaired cell internalization and final tumor uptake of [^99m^Tc]Tc-DT6 during NEP inhibition when compared with the [^99m^Tc]Tc-DT1 reference during double ACE/NEP inhibition [25]. In search of ACE-resistant [^99m^Tc]Tc-DT1 mimics retaining high affinity to the human NTS_1_R as well as favorable internalization capabilities, we developed the DAsn^14^-elongated [^99m^Tc]Tc-DT7 and the β-Homoleucine^13^-substituted [^99m^Tc]Tc-DT8 (Figure 1a,b). Although high receptor affinities were reported for the respective motifs [DAsn^14^-]NT(8-13) [34] and [β-Homoleucine^13^]NT(8-13) [35], in our study, only DT8 retained high NTS_1_R affinity, while DT7 showed considerable affinity loss (Figure 2). On the other hand, both [^99m^Tc]Tc-DT7 and [^99m^Tc]Tc-DT8 achieved increased stability in peripheral mice blood compared with [^99m^Tc]Tc-DT1, which increased by single NEP inhibition (Figure 3, Table 1). Further stabilization could not be achieved by combined NEP/ACE inhibition, a finding implying their becoming resistant to ACE. This assumption is further supported in the case of [^99m^Tc]Tc-DT8 showing no significant improvement of stability during ACE inhibition vs. control. However, receptor specific internalization and uptake in AsPC-1 cells proved to be disappointingly poor for both radioligands, and consequently, they were not considered for further evaluation in tumor-bearing mice.

We subsequently directed our forces to derivatizing [^99m^Tc]Tc-DT1 via the introduction of a pendant ABD handle to enhance metabolic stability. The type and position of this group was carefully selected based on existing reports on lipidation strategies proposed to prolong the bioavailability of peptide ligands [38,39,40,41]. In particular, conjugation of palmitoyl acid at positions Trp^10^ in contulakin-G and Pro^7^ in NT led to improved analogs, thus attracting our attention [42,43,44]. To insert a second functionality in [^99m^Tc]Tc-DT1, the Gly^7^ linker was replaced by Lys^7^, thus offering the *ε*-primary amine for attachment of the palmitoyl group. This modification led to DT9, which, as expected, preserved high affinity binding for the human NTS_1_R (Figure 2). Furthermore, the respective [^99m^Tc]Tc-DT9 radioligand, unlike the C-terminus-modified [^99m^Tc]Tc-DT7 and [^99m^Tc]Tc-DT8, displayed very high internalization capacity in AsPC-1 cells. Notably, internalization values surpassed even that of the [^99m^Tc]Tc-DT1 reference at incubation times longer than 1 h (Figure 3). This finding is in agreement with previous studies showing that lipidation boosts the internalization of peptide analogs [38].

Of particular interest are the results of the in vivo stability study of palmitoylated [^99m^Tc]Tc-DT9, confirming its full resistance to both ACE and NEP (Table 1, Figure 3). This positive outcome was further supported by the fact that treatment of animals with Entresto^®^ (releasing in vivo the potent and selective NEP-inhibitor sacubitrilat [29,30,31]) or the Entresto^®^ plus Lis combination (Lis a potent and selective inhibitor of ACE [26]) had no effect on stability results. It should be emphasized that [^99m^Tc]Tc-DT9 is the first [^99m^Tc]Tc-DT1 mimic that is stable in peripheral mice blood while preserving a high NTS_1_R affinity and high internalization rates in NTS_1_R-expressing cells. These qualities clearly show that the attachment of ABD groups at the linker of NT(8-13) analogs is a promising new strategy.

Next, we evaluated the biodistribution profile of [^99m^Tc]Tc-DT9 in immunosuppressed mice bearing human NTS_1_R-expressing pancreatic cancer xenografts (Table 2, Figure 5). As expected from the stability and internalization results, the tumor uptake of [^99m^Tc]Tc-DT9 was significantly higher than that of unmodified [^99m^Tc]Tc-DT1 at both 4 and 24 h pi. During treatment of mice with the Entresto^®^ plus Lis combination, tumor uptake was improved only for the biodegradable [^99m^Tc]Tc-DT1 reference but not for [^99m^Tc]Tc-DT9, once again confirming the successful in vivo targeting of the palmitoylated mimic. However, the overall pharmacokinetic profile of [^99m^Tc]Tc-DT9 turned out to be suboptimal, mainly due to high radioactivity levels found in the blood. High radioactivity levels in the blood resulted in elevated background radioactivity, especially in the liver and intestines, as previously reported for other albumin-binding lipophilic compounds [51]. It is evident that palmitoyl does not represent the ABD group of choice for Lys^7^ attachment, but [^99m^Tc]Tc-DT1 mimics derivatized from different groups may prove to be appropriate candidates for clinical translation. 

## 5. Conclusions

Aiming at [^99m^Tc]Tc-DT1 mimics for combined resistance to ACE and/or NEP with high NTS_1_R affinity and high internalization rates, we followed two major strategies. Firstly, we developed the C-terminus-modified DT7 ([DAsn^14^]DT1) and DT8 ([β-Homoleucine^13^]DT1) and, secondly, the palmitoyl-decorated DT9 ([(palmitoyl)Lys^7^]DT1). During head-to-head comparisons in NTS_1_R-expressing cells, only [^99m^Tc]Tc-DT9 achieved high internalization rates. It also proved to be the only radioligand stable in peripheral mice blood, displaying resistance to both ACE and NEP. In mice bearing NTS_1_R-expressing pancreatic cancer xenografts, [^99m^Tc]Tc-DT9 showed significantly higher tumor uptake compared with the [^99m^Tc]Tc-DT1 reference, while during twin ACE/NEP inhibition, uptake was comparable between these analogs. The high background activity levels of [^99m^Tc]Tc-DT9 in mice, however, led to an unfavorable pharmacokinetic profile. In conclusion, coupling of an ABD group on Lys^7^ in [^99m^Tc]Tc-DT1 mimics showed to be the preferred approach toward metabolic stability, cell internalization, and tumor targeting compared with the hitherto less successful C-terminal modification. Replacement of palmitoyl by other ABD groups to improve pharmacokinetics is warranted and is currently being pursued. 

## Figures and Tables

**Table 1:** Stabilities of [^99m^Tc]Tc-DT1, [^99m^Tc]Tc-DT7, [^99m^Tc]Tc-DT8, and [^99m^Tc]Tc-DT9 in peripheral mouse blood 5 min pi without treatment with NEP/ACE inhibitor(s) (controls) or treated with Entresto^®^ or Lis or their combination (Entresto^®^+Lis).

	[^99m^Tc]Tc-DT1 ^1^	[^99m^Tc]Tc-DT7	[^99m^Tc]Tc-DT8	[^99m^Tc]Tc-DT9
Control	1.81 ± 0.77 (n = 4)	26.91 ± 1.91 (n = 3)	20.68 ± 3.10 (n = 3)	98.06 ± 1.18 (n = 3)
Entresto^®^	5.46 ± 3.86 (n = 5)	56.61 ± 7.92 (n = 6)	60.72 ± 8.35 (n = 3)	97.33 ± 1.7 (n = 3)
Lis	18.77 ± 2.54 (n = 3)	-	28.82 ± 4.59 (n = 3)	-
Entresto^®^+Lis	63.80 ± 7.51 (n = 3)	60.27 ± 11.82 (n = 3)	64.06 ± 4.07 (n = 3)	93.72 ± 3.7 (n = 3)

^1^ Metabolic stability results for [^99m^Tc]Tc-DT1 have been adapted from [24,25]; data represents the mean percentage of intact radioligand ± sd; number of experiments are shown in parentheses.

**Table 2:** Comparative biodistribution data of [^99m^Tc]Tc-DT9 in SCID mice bearing AsPC-1 xenografts at 4 h (block, controls, and Entresto^®^+Lis treated) and 24 h pi (controls and Entresto^®^+Lis treated); data are expressed as %IA/g and represent average values ± sd, n = 4.

Organs/Tissues	[^99m^Tc]Tc-DT9				
	4 h			24 h	
	Block	Controls	Entresto^®^+Lis	Controls	Entresto^®^+Lis
Blood	4.25 ± 0.37	4.63 ± 0.55	4.32 ± 0.42	0.68 ± 0.18	0.70 ± 0.06
Liver	30.93 ± 4.25	29.56 ± 3.77	28.42 ± 2.48	12.93 ± 1.66	16.96 ± 0.94
Heart	2.79 ± 0.47	3.12 ± 0.43	2.85 ± 2.48	0.64 ± 0.11	0.71 ± 0.06
Kidneys	8.56 ± 0.87	9.53 ± 1.18	9.15 ± 0.62	3.81 ± 0.54	4.63 ± 0.15
Stomach	2.06 ± 0.47	3.09 ± 1.06	2.27 ± 0.38	1.73 ± 0.50	1.56 ± 0.31
Intestines	6.27 ± 0.72	13.02 ± 4.60	7.90 ± 0.88	4.26 ± 0.61	5.37 ± 1.61
Spleen	6.18 ± 1.37	6.82 ± 1.11	5.47 ± 0.47	3.42 ± 0.54	4.47 ± 0.83
Muscle	0.82 ± 0.11	0.93 ± 0.13	0.81 ± 0.04	0.22 ± 0.02	0.24 ± 0.01
Lungs	8.46 ± 1.26	8.80 ± 1.42	7.61 ± 0.64	3.42 ± 0.67	3.62 ± 0.94
Pancreas	1.84 ± 0.41	2.10 ± 0.30	1.85 ± 0.18	0.75 ± 0.17	0.80 ± 0.07
AsPC-1 Tumor	3.68 ± 0.92	6.15 ± 0.92	5.24 ± 0.27	3.32 ± 0.35	2.84 ± 0.43

## Data Availability

Data is contained within the article or Supplementary Material.

## Supplementary Materials

The following supporting information can be downloaded at: https://www.mdpi.com/article/10.3390/pharmaceutics15082092/s1, Table S1: Analytical data for DT7, DT8, and DT9.
