# Supplementary material for: Toward Stability Enhancement of NTS1R-Targeted Radioligands: Structural Interventions on [99mTc]Tc-DT1
Source: Pharmaceutics. 2023 Aug 7;15(8):2092. doi: 10.3390/pharmaceutics15082092 (PMC10459693; doi:10.3390/pharmaceutics15082092)
Supplement: Supplementary file 1 [file pharmaceutics-15-02092-s001.zip › pharmaceutics-2545900-supplementary.pdf]

pharmaceutics

Article

Supplementary Materials: Toward Stability Enhancement of NTS_1R-Targeted Radioligands: Structural Interventions on $[^{99m}Tc]Tc$-DT1

Panagiotis Kanellopoulos [1], Berthold A. Nock [1], Eric P. Krenning [2] and Theodosia Maina [1,*]

Analytical data for DT7, DT8 and DT9

Analytical data for the DT1 (N_4-Gly[7]-Arg-Arg-Pro-Tyr-Ile-Leu-OH; N_4, 6-(carboxy)-1,4,8,11-tetraazaundecane) mimics DT7 ([DAsn[14]]DT1), DT8 ([β-Homoleucine[13]]DT1) and DT9 ([(palmitoyl)Lys[7]]DT1) from PiChem Forschungs- und Entwicklungs GmbH (Raaba-Grambach, Austria), comprising purity via HPLC analysis and MALDI-TOF data is summarized in Table S1.

Table S1. Analytical data for DT7, DT8 and DT9.[a]

	HPLC				MW [d] calcd	MW found [e], m/z
	t_R (min)		% Purity			
DT7	13.7 [a]	17.1 [c]	>95 [a]	>99 [c]	1174.4	1175.2
DT8	8.7 [b]	19.1 [c]	>95 [b]	>99 [c]	1076.3	1076.2
DT9	19.2 [a]	37.7 [c]	>90 [a]	>99 [c]	1367.8	1368.8

[a] A Nucleosil C18 (5 µm, 4 mm × 150 mm) column (MACHEREY-NAGEL GmbH & Co. KG; Dueren, Germany) was eluted at 1 mL/min flow rate with the following gradient: 90%A/10%B to 50%A/50%B in 30 min, UV trace at 215 nm, while in [b] 90%A/10%B to 10%A/90%B in 50 min was applied; A: 0.1% TFA, B: 0.1%TFA in MeCN. In an additional analysis system, a Waters Symmetry Shield RP-18 (5 µm, 4.6 mm × 150 mm) cartridge column (Waters, Vienna, Austria) was eluted at a 1 mL/min flow rate with the following linear gradient: from 100%A/0%B to 40%A/60%B in 60 min; A = 0.01% TFA and B = MeCN – UV trace at 220 nm; [d] average mass; [e] verification on MALDI TOF mass spectrometry.
